# Prognostic factors in the myoepithelial-like spindle cell type of metaplastic breast cancer

**DOI:** 10.1007/s00428-016-1950-9

**Published:** 2016-05-25

**Authors:** Fabian Leo, Stephan Bartels, Lavinia Mägel, Theodor Framke, Guntram Büsche, Danny Jonigk, Matthias Christgen, Ulrich Lehmann, Hans Kreipe

**Affiliations:** 1 Institute of Pathology, Hannover Medical School, Carl-Neuberg-Str. 1, 30625 Hannover, Germany; 2 Institute of Biometry, Hannover Medical School, Carl-Neuberg-Str. 1, 30625 Hannover, Germany

**Keywords:** Breast cancer, Metaplastic, Myoepithelial, Prognosis, PIK3CA

## Abstract

Metaplastic breast carcinoma (MBC) comprises a heterogeneous group of tumors with difficult to predict biological behavior. A subset of MBC, characterized by spindle-shaped tumor cells with a myoepithelial-like immunophenotype, was entered into a retrospective study (*n* = 42, median follow-up time 43 months). Molecular parameters (DNA sequences of mutation hot spots in AKT1, ALK, APC, BRAF, CDH1, CTNNB1, EGFR, ERBB2, FBXW7, FGFR2, FOXL2, GNAQ, GNAS, KIT, KRAS, MAP2K1, MET, MSH6, NRAS, PDGFRA, PIK3CA, PTEN, SF3B1, SMAD4, SRC, SRSF2, STK11, TP53, and U2AF1; copy numbers for EGFR, c-myc, FGFR, PLAG, c-met) were assessed. None of the patients had axillary lymph node involvement. In 13 cases, local recurrence developed after surgery (30.9 %). Distant metastasis occurred in seven patients (17 %; four after local recurrence). The most frequent genetic alteration was PIK3CA mutation (50 % of cases). None of the pathological parameters (size, grade, stage, Ki-67 labeling index) was significantly associated with disease-free survival (DFS) or overall survival (OS). PIK3CA mutation, especially the H1047R type, tended to adversely affect OS. Type of resection (mastectomy vs. breast-conserving therapy, width of margins) or adjuvant radiotherapy had no influence on DFS or OS, whereas in the group treated with radio-/chemotherapy, no local recurrence or metastasis and no death occurred. We conclude that the spindle cell type of MBC with myoepithelial features exhibits a higher frequency of PIK3CA mutation than other types of metaplastic or basal-like breast cancer and may benefit from combined radio-/chemotherapy. Classical pathological parameters are not helpful in identifying the high-risk tumors among this subgroup of MBC.

## Introduction

Whereas prospective studies have identified prognostic factors in invasive breast carcinoma of no special type, knowledge is limited in the rare special types. Metaplastic breast carcinomas (MBCs) comprise a heterogeneous group of neoplasms. Whereas some MBCs are clearly high-grade tumors and are easily recognizable as malignant, a considerable proportion of spindle cell MBC can appear deceptively benign. These tumors are often misdiagnosed as nodular fasciitis, fibromatosis, and scarring reaction on the one hand or as low-grade sarcomas or fibrosarcomas, on the other [1, 2]. This subset of MBC is composed of neoplastic spindle cells with an immunophenotype identical to myoepithelial cells [3–6]. Spindle cell myoepithelial carcinoma (SCMC) is difficult to distinguish from other spindle cell mammary neoplasms by light microscopy, and in most instances, immunohistochemistry is required for its recognition. Many descriptive terms have been used to classify this particular type of MBC, such as matrix-producing carcinoma, carcinosarcoma, spindle cell carcinoma, and carcinoma with pseudosarcomatous metaplasia, with metaplastic spindle cell or sarcomatoid breast cancer being the most widely applied designations. The new World Health Organization (WHO) classification (2012) subdivides the rubric of MBC of spindle cell type into fibromatosis-like metaplastic carcinoma (8572/3), spindle cell carcinoma (8032/3), and myoepithelial carcinoma (8982/3) [7]. These lesions are considered to constitute a continuum without definite criteria for discrimination.

Because the term MBC encompasses a group of diverse tumors, it has been difficult to reliably predict biological potential or to determine optimal therapy [8]. Despite their benign appearance, spindle cell carcinomas of the breast have been reported to exhibit aggressive behavior with a tendency to progression similar to invasive duct carcinomas [9]. Histological features such as grade, cellularity, mitotic activity, differentiation of the carcinoma, presence of squamous epithelium, and degree of inflammation were not found to predict outcome [10]. Including the cytologically bland fasciitis-like variant, spindle cell MBCs were found by some investigators to be highly aggressive neoplasms with a high rate of extra-nodal metastases [8, 11, 12], although they may have a significantly lower rate of nodal metastases than conventional ductal or lobular carcinomas [8, 11, 13]. Other studies reported that the spindle cell phenotype appears to behave less aggressively when compared to the whole group of MBC [13], and no distant metastasis was found in a small series of 10 cases [14].

Up to now, only little is known about the somatic mutations which give rise to SCMC. Studies are hampered by the heterogeneity of MBC and only rarely have correlations of molecular findings with the histologic subtypes within this rubric of mammary neoplasms been undertaken. Biphasic and monophasic types of MBC have been lumped together [15]. Accordingly, it was found that MBC constitutes a heterogeneous group of tumors also in terms of their gene copy number aberrations and transcriptomic profiles [16]. Genetic changes of Wnt pathway genes have been found as common events in MBC [17]. This could not be confirmed by Lacroix-Triki et al. [18] who identified no *CTNNB1* mutation in any of the 21 MBCs. A high copy number of *EGFR* via aneusomy has been described [19]. Overexpression of EGFR by MBC is partly caused by gene amplification without evidence of activating mutations within the *EGFR* gene [20]. Frequent aberrations of the *p53* tumor suppressor gene were found to characterize MBC [15, 21]. In the case of bimodal differentiation, both components shared *p53* mutation indicating that the gene is altered at an early stage of clonal expansion [15, 21].

In this study, we focused on one special subtype of MBC which is composed of spindle cells, demonstrating matrix production and a myoepithelial immunophenotype. Recently, we could show that these types of cancer reveal a miRNA profile which is identical to normal myoepithelial cells whereas non-spindle cell carcinomas with expression of basal markers resembled luminal cells [22]. Non-spindle so called basal carcinomas and carcinosarcomas with an accompanying invasive adenocarcinoma component were excluded from the study.

## Patients, materials, and methods

All cases in this retrospective study (*n* = 42) were sent to the reference center for breast pathology in Hannover, Germany, for histopathological consultation between 1999 and 2014. Tumors composed of a predominant spindle cell population with matrix production featuring a myoepithelial immunophenotype (positive for cytokeratins 5/14 and/or p63/p40 in at least 10 % of tumor cells) and infiltrative growth were included. Non-infiltrative lesions with myoepithelial characteristics, such as adenomyoepithelioma, and malignant tumors featuring invasive glandular components or heterogeneous elements except for foci of squamous differentiation were excluded. Representative paraffin blocks were used for all further studies. Tissue from metastases was available in three cases and from local recurrences in four cases, respectively. Clinical information was retrospectively gathered from the requesting hospitals and the patients’ general practitioner or registered gynecologist. Written informed consent was obtained from all patients entering the study, or their respective family members or legal guardian. The study was confirmed by the Ethic Committee of Hannover Medical School.

Patient (age, menopausal status) and tumor (size, nodal status, metastasis) characteristics as well as types of treatment (breast-conserving surgery vs. mastectomy, width of margins) and adjuvant therapies (chemotherapy, radiotherapy) were assessed. The clinical course was evaluated with regard to disease-free (occurrence of local recurrence and distant metastasis) and overall survival. Mean follow-up interval was 43 months (range 9 to 167 months). One patient known to have a co-existing malignant disease at the time of diagnosis was excluded from follow-up evaluation.

### Immunophenotyping

For immunohistochemistry, 2-μm sections of formalin-fixed paraffin-embedded (FFPE) tumor specimens were mounted on poly-L-lysine-coated slides and deparaffinized and rehydrated conventionally. Immunohistochemical stainings were performed on a Benchmark Ultra (Ventana, USA) automated stainer using the CC1-mild or CC1-st protocols for target retrieval and the anti-CD10 (clone 56C6, Thermo Scientific, Germany, 1:10), anti-CD34 (clone QBEnd/10, Leica Biosystems, Germany, 1:50), anti CK5/14 (clone XM26/LL002, Zytomed Systems, Germany, 1:100), anti-CK8/18 (clone 5D3, Leica Biosystems, 1:100), anti-p63 (clone Y4A3, Zytomed Systems, 1:50), anti-p40 (rabbit polyclonal, PC373, Calbiochem, U.S.A., 1:1000), anti-smActin (clone 1A4, Dako, Denmark, 1:100), anti-EGFR (clone 2-1E1, Zytomed Systems, 1:200), anti-ER (clone SP1, Ventana, 1:1), anti-PR (clone 1E2, Ventana, 1:1), anti-HER2 (clone 4B5, Ventana), and anti-Ki-67 (clone SP6, Thermo Scientific, 1:100) antibodies. Besides characteristic histomorphology, at least two of the three myoepithelial markers (CK5/14, p63 or p40, CD10) being positive in more than 10 % of tumor cells were required for inclusion into the study.

### Fluorescence in situ hybridization

Representative formalin-fixed paraffin-embedded cores (2 per case, each 1.4mm^2^) from each of the tumors (*n* = 42) were built into a tissue microarray (TMA) for fluorescence in situ hybridization (FISH). FISH studies were performed following standard protocol on 3-μm-thick unstained sections, mounted on Super Frost Plus slides (Menzel, Germany). Slides were dried in a Heraeus dry incubator (ThermoScientific) at 60 °C for 1 h and subsequently pretreated by dewaxing with xylene supplementation (AppliClear, AppliChem, Germany), followed by rehydration in ethanol in descending concentrations (100, 85, and 70 %). Specimens were then permeabilized and denatured in a microwave oven for 30 min with sodium citrate buffer (pH 6.0) (Sigma Aldrich, USA), cooled to room temperature, and rinsed in distilled water. This was followed by incubation with a pepsin solution for 15 min at 37 °C and subsequently passed through ascending concentration of ethanol (70, 80, and 100 %). Hybridization was started by using different FISH probes to analyze for abnormalities of the genes *c-myc*, *c-met*, *EGFR*, *FGFR1* [23], and *PLAG1* [24] (ZytoLight SPEC CMYC/CEN8 Dual Color Probe, ZytoVision, Germany; ZytoLight SPEC CMYC/CEN8 Dual Color Break Apart, ZytoVision; ZytoLight SPEC MET/CEN7 Dual Color Probe, ZytoVision; ZytoLight SPEC EGFR/CEN7 Dual Color Probe, ZytoVision; ZytoLight SPEC FGFR1/CEN8 Dual Color Probe, ZytoVision and PLAG1 FISH probe, Abnova). Specimens were heated to 80 °C for 10 min to denature the probe and specimen DNA and incubated overnight at 37 °C in a ThermoBrite slide processing system (Abbott). Subsequently, specimens were washed in 0.4×SSC (sodium chloride sodium citrate, Abbott) at 75 °C for 2 min and passed through ascending concentrations of ethanol (70, 85, and 100 %). Finally, slides were counterstained with DAPI (4′,6-diamidin-2-phenylindol) 40 ng/ml (Qiagen, Netherlands). At least 100 non-overlapping interphase tumor cell nuclei with hybridization signals were evaluated for each case with a fluorescence microscope (Olympus BX51) at a ×630/1000 magnification (oil immersion objective). Thresholds for aberrant counts were defined for each probe according to the manufacturer’s specifications and our established procedure.

### DNA sequencing

From tumor-bearing paraffin blocks, 5-μm-thick sections were cut and tumor infiltrates were enriched by microdissection. From each specimen, two to six sections were taken, depending on tumor size. Genomic DNA was extracted from FFPE specimens with DNeasy Blood & Tissue Kit (Qiagen) according to the manufacturer’s recommendations. DNA quantification was performed using the Qubit 2.0 Fluorometer with dsDNA high sensitivity Assay kit (Life Technologies). In total, DNA from 36 patients was available and in an amount and quality adequate for sequencing. For initial screening of the study cohort, 13 samples were analyzed with the Illumina TruSight™ Tumor Sequencing Panel (Illumina). Median of the mean sequencing depth for these 13 patient samples was 1506 reads (range 175–4885). To validate results, six of these samples plus additional 23 patients were analyzed with Ion AmpliSeq™ Colon and Lung Cancer Research Panel v2 with a median of mean sequencing depth of 3185 reads (range 819–6595). The TruSight™ Tumor Sequencing Panel (Illumina) comprises 175 amplicons of 26 genes (*AKT1*, *ALK*, *APC*, *BRAF*, *CDH1*, *CTNNB1*, *EGFR*, *ERBB2*, *FBXW7*, *FGFR2*, *FOXL2*, *GNAQ*, *GNAS*, *KIT*, *KRAS*, *MAP2K1*, *MET*, *MSH6*, *NRAS*, *PDGFRA*, *PIK3CA*, *PTEN*, *SMAD4*, *SRC*, *STK11*, and *TP53*) with relevance in different types of cancer. Amplicon size was between 165 and 196 Bp. Amplicon-based library preparation requires 100 ng purified genomic DNA. Sequencing was performed on a MiSeq Instrument with MiSeq Reagent Kit v2 (300 cycles) (Illumina) with 10 samples on a flow cell. The Ion AmpliSeq™ Colon and Lung Cancer Research Panel v2 (Life Technologies) comprises 92 amplicons from the hotspot regions of 19 genes (*AKT1*, *ALK*, *BRAF*, *CTNNB1*, *DDR2*, *EGFR*, *ERBB2*, *ERBB4*, *FBXW7*, *FGFR1*, *FGFR2*, *FGFR3*, *KRAS*, *MAP2K1*, *MET*, *NOTCH1*, *NRAS*, *PIK3CA*, *PTEN*, *STK11*, and *TP53*) which are well-described to be of relevance in different types of cancer, including breast cancer [23, 25, 26]. The panel is designed for FFPE DNA and requires 10 ng of input material. Amplicon target region lengths were between 51 and 137 Bp. Library preparation was performed with the Ion AmpliSeq Library Kit 2.0 (Life Technologies). Quantitation of the libraries was conducted by qPCR with the Ion Library Quantitation Kit (Life Technologies). For template preparation in the Ion OneTouch 2 instrument (Life Technologies), eight patient samples were pooled (100 pM each). Sequencing was performed on the Personal Genome Machine (PGM) with Ion PGM Sequencing 200 Kit v2 on 316 v2 chips (all Life Technologies). Analyses of sequencing raw data were performed with Torrent server software version 4.2.1 (Life Technologies), IGV-Browser Version 2.3.34 (http://broadinstitute.org/igv/), and Cartagenia Bench Lab NGS software version 3.1.2 (Cartagenia). Parameters for analysis exclude single nucleotide variants with an allele frequency <2 % and complex mutations with an allele frequency <5 %, and a quality score (PHRED-scaled probability of incorrect calls) below 100. In addition, the study cohort was analyzed by Sanger sequencing on GenomeLab™ GeXP capillary sequencer (Beckman Coulter) to verify variants reported from targeted next-generation sequencing (NGS) and to analyze splice factor genes [26]. Sanger sequencing was performed on exon-coding sequences of *PIK3CA* (exons 1, 3, 7, 8, 9, and 20), *TP53* (exons 5–8), *SRSF2* (exon 1), *U2AF1* (exons 2 and 6), and *SF3B1* (exons 14 and 15) [27]. PCR amplification of DNA was done in a total volume of 25 μl PCR mix containing 10–50 ng template DNA, Taq buffer, 2.5 mM MgCl2, 200 μmol of each deoxynucleotide triphosphate, 10 pmol of each primer, and 0.5 U of Invitrogen™ Platinum Taq (Life Technologies). PCR amplification conditions were as follows: 95 °C 10 min; 95 °C 30 s, 60 °C 45 s, 72 °C 30 s for 40 cycles; 72 °C 10 min. The sequence data files were analyzed with SeqMan Pro software version 8.1.4 (DNASTAR®).

### Statistical methods

A survival analysis was carried out to describe clinical outcome and to identify possible variables with prognostic value. Time from surgery to death due to any cause was considered of primary interest. In addition, disease-free survival (defined as time from surgery to occurrence of local recurrence or distant metastasis) was evaluated. Patients were censored at the time of their last contact with the investigators in case of no event. Kaplan-Meier plots were used to display the course of survival, and the log-rank was calculated to test for differences of the survival functions. Based on the Kaplan-Meier estimates, the median survival time and its 95 % confidence interval were calculated if possible. The mean ± SE was also calculated; however, the mean values may be biased downward as the censoring time was greater than the largest observed event time.

A Cox PH model was set up for overall and disease-free survival. The proportional hazard assumption was checked via the cumulative sums of martingale residuals and the calculation of Schoenfeld residuals. In a first step, we assessed the impact of each of the covariables on the survival time separately. Additionally, we tried to find a suitable fractional polynomial with the help of the %mfp8 macro (available from https://portal.uni-freiburg.de/imbi/mfp/) to check for a possible non-linear relationship. Subsequently, a multiple regression model was set up involving variables considered of importance based on the univariate analysis. In this model, a backward-selection approach was applied to remove non-informative variables. Due to the explorative nature of this study, no multiplicity adjustments were performed, and thus, the type I error rate could not be controlled.

The statistical analysis was carried out with SAS 9.3 software (SAS Institute Inc., USA).

## Results

### Patient and tumor characteristics and outcome

Forty-two patients, aged 39–92 years (median 74 years), were included in the study. All patients had presented with a palpable, singular nodule of the breast. Tumor size was known in 41 cases and varied from 5 to 85 mm (median 30 mm). One tumor showed infiltration of the pectoral muscle, and all other lesions were confined to the breast and were categorized as pT1–pT3 according to the TNM classification of breast tumors (pT1 ≤ 20 mm; *n* = 11, pT2 > 20–50 mm; *n* = 24, pT3 > 50 mm; *n* = 6). No patient had lymph node involvement, as assessed either by sentinel node examination (*n* = 20), axillary dissection (*n* = 11), or ultrasound imaging (*n* = 10). No patient had evidence of distant metastasis at time of diagnosis. Consequently, all patients were treated surgically as a curative approach.

**Fig. 1:**
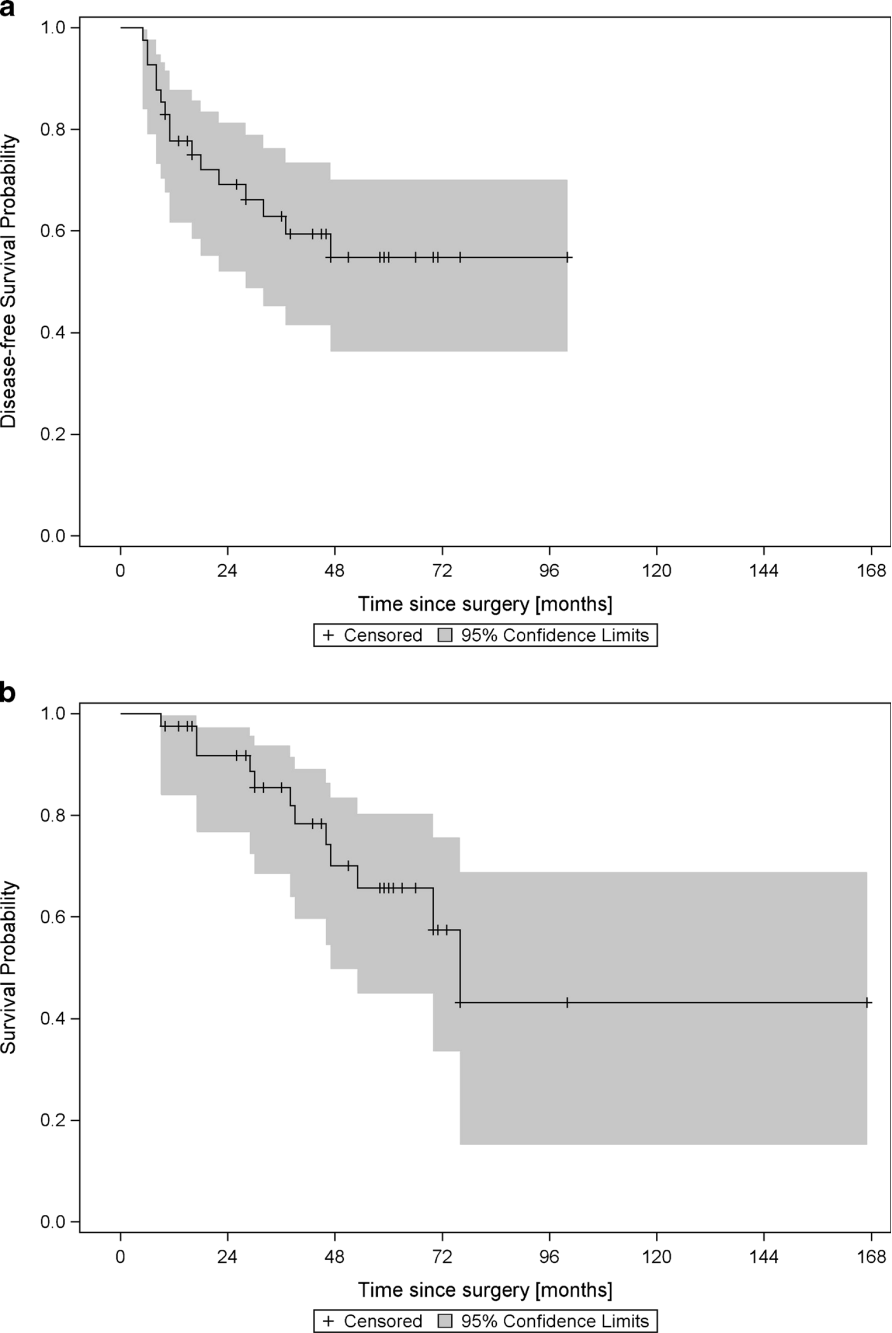
Kaplan-Meier plots of disease-free survival (**a**) and overall survival (**b**) in a cohort of 41 patients with spindle cell myoepithelial carcinomas (SCMCs)

Forty-one patients could be evaluated. Median time of follow-up was 43 months (range 9–167 months). Median survival time was 76 months (mean ± SE, 61.4 ± 3.9 months) for overall survival. For disease-free survival, a mean time of 34.5 ± 2.7 months was observed. Within the observed time period, 12 (29.3 %) patients died due to any cause, and in 16 patients (39.0 %) relapses were noted (Fig. 1a, b). Nine patients (21.4 %) had local recurrence but did not develop distant metastasis. After a median follow-up of 5 years (61 months, range 16–167 months) after the initial procedure, all nine patients with local recurrence were alive with no evidence of disease. Local recurrence occurred after a median time of 10 months (range 5–37 months), and all patients received surgical revision. One patient had another tumor relapse after 11 months, but remained disease-free after another resection during the 22-month follow-up. Seven patients (17.1 %) developed distant metastasis with or without (*n* = 3) prior or simultaneous diagnosis of local recurrence. Median time until development of distant metastasis was 16 months (6–47 months). All patients in this group died within a period of 9–53 months after the initial operation (median 29 months). The most common sites of distant metastasis were lungs, bones, and liver (Table 1).

**Table 1:** Sites of metastasis

Case	Type of surgery	Resection margin	Tumor size	Nuclear grading	Ki-67 index	Sites of metastasis	RFS (months)	OS (months)	Adjuvant therapy
1	Mastectomy	25 mm	30 mm	G2	15 %	Soft tissue (mediastinum), myocardium, pleura	46	52	None
2	BCT	3 mm	20 mm	G1	15 %	Lungs	16	17	Radiotherapy
3	BCT	N.A.	35 mm	G2	20 %	Lungs	11	17	None
4	Mastectomy	N.A.	42 mm	G2	10 %	Lungs, liver	22	30	None
5	Mastectomy (secondary)	N.A.	45 mm	G2	20 %	Brain, lungs, liver, bone	18	29	None
6	Mastectomy	20 mm	60 mm	G2	40 %	Lungs, adrenal	5	7	Radiotherapy
7	Mastectomy	5 mm	18 mm	G2	20 %	Lungs, bone, soft tissue (thorax)	9	39	None

*BCT* breast-conserving therapy, *N.A.* not assessed

### Correlation of clinical and pathological parameters with outcome

DFS and OS did not differ significantly in patients <74 years compared to patients 74 years and older (Table 2). Tumors ≤20 mm (pT1) had a lower rate of local recurrence, metastasis, and death compared to tumors >20 mm (pT2, pT3) (hazard ratio 2.82 for DFS and 3.28 for OS for tumors >20 mm), but the difference was not statistically significant. There were also no significant differences when tumors ≤50 mm (pT1, pT2) were compared to tumors >50 mm (pT3) (Table 2).

**Table 2:** Patient data and outcome

Parameter		Disease-free survival [months]			Overall survival [months]		
		Mean ± SE	Log-rank test	HR (95 % CI)	Mean ± SE	Log-rank test	HR (95 % CI)
Age Range, 39–92 years Median, 74 years	<74 years^(1)^ (*n* = 21)^(2)^ ≥74 years (*n* = 20)^(2)^	26.0 ± 3.0 38.3 ± 3.7	0.3156	0.60 (0.20, 1.62)	35.4 ± 2.0 60.7 ± 5.4	0.4076	1.62 (0.51, 5.48)^(5)^
Tumor size Range, 5–85 mm Median, 30 mm	≤50 mm^(1)^ (*n* = 34)^(2)^ >50 mm (*n* = 6)^(2)^	34.7 ± 2.9 6.0 ± 0.0	0.7194	1.31 (0.20, 4.77)	58.9 ± 3.6 40.7 ± 8.2	0.3578	2.03 (0.31, 8.00)
	≤20 mm^(1)^ (*n* = 10)^(2)^ >20 mm (*n* = 30)^(2)^	34.1 ± 3.9 31.6 ± 3.4	0.1505	2.82 (0.79, 17.97)	39.0±– 55.2 ± 4.3	0.2294	3.28 (0.63, 60.25)
Grading	G1^(1)^ (*n* = 10)^(2)^	29.2 ± 4.4	0.9536^(3)^		69.4 ± 8.7	0.4802^(3)^	
	G2 (*n* = 23)^(2)^	34.5 ± 3.8	0.7818	1.16 (0.38, 4.30)	45.4 ± 2.9	0.2424	2.60 (0.61, 17.77)
	G3 (*n* = 8)^(2)^	10.3 ± 0.7	0.9856	0.99 (0.20, 4.49)	57.1 ± 9.6	0.3791	2.22 (0.37; 16.93)
Ki67%	≤20^(1)^ (*n* = 19)^(2)^	30.2 ± 4.1	0.0604		57.3 ± 5.6	0.1951	
	>20 (*n* = 22)^(2)^	27.7 ± 2.2		0.51 (0.19, 1.44)	64.2 ± 4.7		1.49 (0.46, 5.69)
PIK3CA	No mutation (*n* = 18)^(2)^	34.4 ± 3.8	0.9397		60.0 ± 5.0	0.2413	
	Mutation (*n* = 18)^(2)^	13.5 ± 1.1		0.96 (0.32, 2.68)^(5)^	41.1 ± 3.4		2.16 (0.57, 8.82)
	No mutation (*n* = 24)^(2)^	35.1 ± 3.4	0.5564		59.9 ± 4.3	0.1447	
	H1047R mutation (*n* = 12)^(2)^	13.2 ± 1.3		1.38 (0.43, 3.96)^(5)^	38.4 ± 4.6		2.59 (0.63, 9.96)
Surgery	BCT^(1)^ (*n* = 23)^(2)^	28.0 ± 2.8	0.7733		61.6 ± 4.2	0.1909	
	Mastectomy (*n* = 18)^(2)^	35.9 ± 4.5		0.86 (0.29, 2.33)	55.9 ± 6.8		2.12 (0.67, 7.20)
	Margin <5 mm (*n* = 16)^(2)^	27.6 ± 3.4	0.7707		44.8 ± 2.5	0.4963	
	Margin ≥5 mm (*n* = 22)^(2)^	32.3 ± 5.7		1.18 (0.38, 3.54)	47.2 ± 4.7		1.73 (0.32, 9.42)
Adjuvant therapy	None^(1)^ (*n* = 22)^(2)^	31.0 ± 3.8	0.0507^(3)^		57.6 ± 5.3	0.0715^(3)^	
	Radiotherapy (*n* = 10)^(2)^	14.0 ± 1.5	0.1715	0.62 (0.14, 1.97)	57.1 ± 9.1	0.4120	0.82 (0.18, 2.85)
	Radio−/chemoth. (*n* = 7)^(2)^	N.E.	0.0154	N.E.	N.E.	0.0790	N.E.

(1) = Reference category chosen for the Cox PH model, (2) = case number, (3) = global. Median survival was calculated according to the Kaplan-Meier method. Calculation of median survival and its upper or lower confidence interval limits or SE were not always possible. These cases are indicated as (–). There were no events in the Radio−/Chemotherapy group (N.E.). In some models, the Cox PH assumption may not be met (5). For variables with 3 groups, a global log-rank test was performed as well as pairwise comparisons with a reference

*CI* confidence interval, *HR* hazard ratio, *BCT* breast-conserving therapy, *SE* standard error

**Fig. 2:**
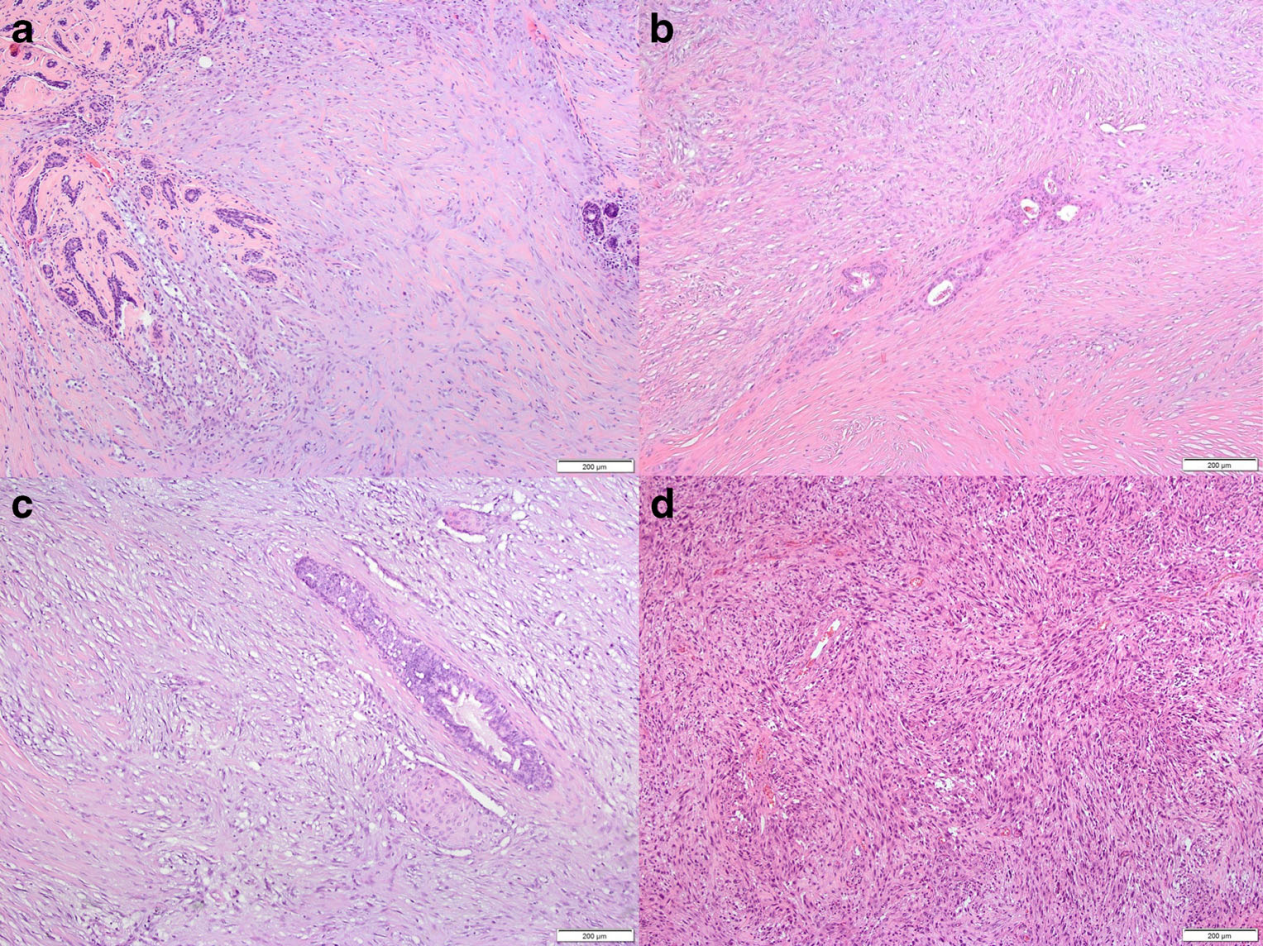
Spindle cell myoepithelial carcinomas (SCMCs) were characterized by proliferation of spindle cells with slender nuclei in varying densities, infiltrating around pre-existing ducts and lobuli (**a**–**c**). All tumors exhibited matrix production but to varying extents (**a**, **b**). Due to mucoid change of intercellular matrix, pseudoangiomatoid spaces occurred in some cases (**a**, *lower left quadrant*) with an endothelial-like inner cell layer around the mucoid material. Occasionally, foci of squamous differentiation not exceeding 10 % of tumor cells were found (**c**). Brain metastasis retained spindle cell morphology but revealed a higher cell density (**d**) (**a**–**d**, hematoxylin-eosin stain)

**Fig. 3:**
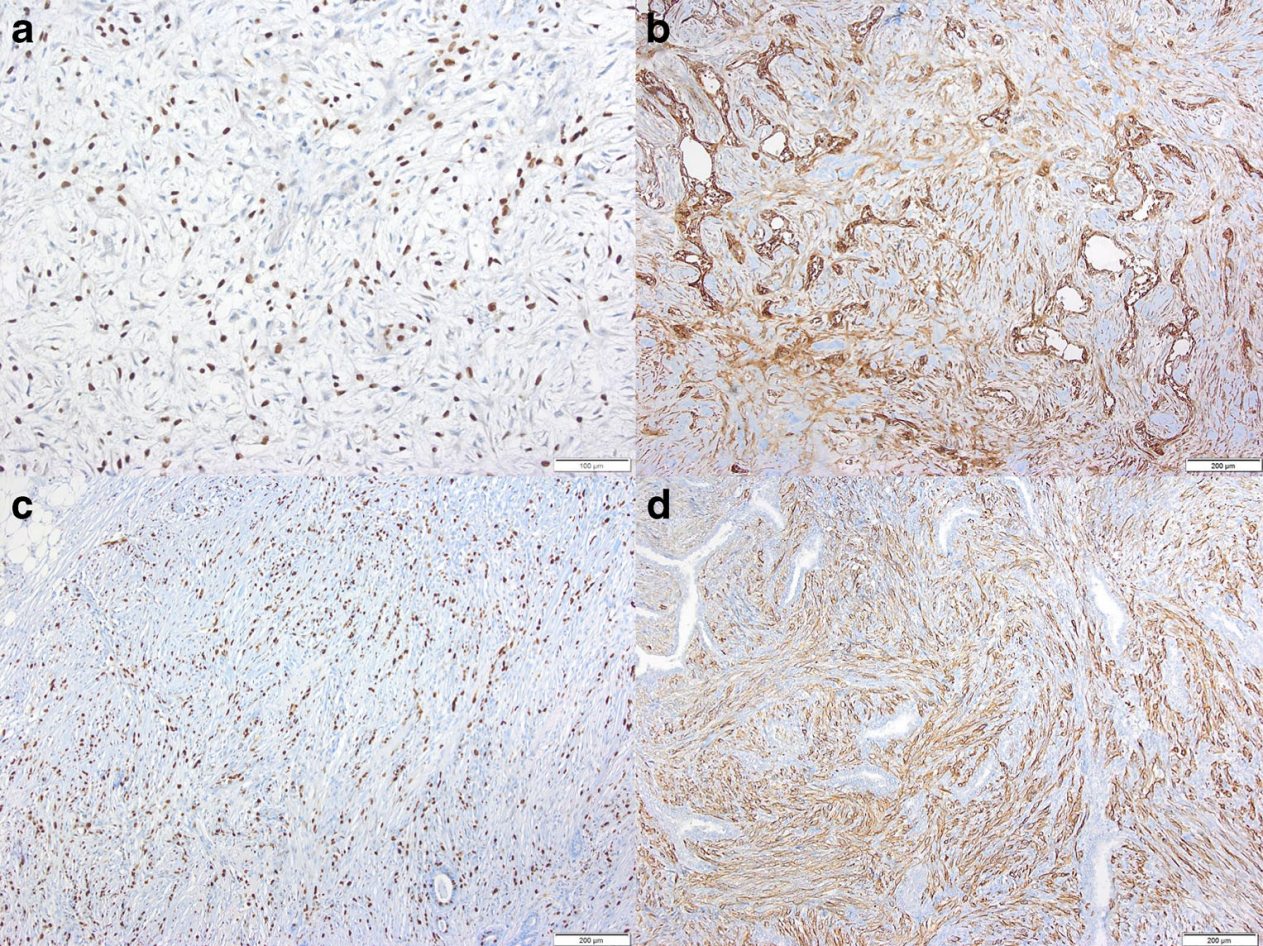
The myoepithelial marker P63 was present in the nuclei of spindle cells in SCMC (**a**). CD10 was expressed in nearly all cases whereby spindle cells as well as endothelial-like cells around cystic spaces exhibited positive labeling (**b**). Despite the bland morphology of spindle cell proliferates in SCMC, most cases showed a Ki-67 labeling index exceeding 10 % (**c**). High molecular weight cytokeratin (5, 14), which characterizes myoepithelial cells, was expressed by SCMT including metastasizing cases (**d**, lung metastasis) (**a**–**d**, myeloperoxidase immunohistology)

All cases were characterized by proliferation of spindle cells with slender nuclei in varying densities. Typically, the spindle cells infiltrated around pre-existing ducts and lobuli (Fig. 2a–c). Matrix production was heterogeneous within single tumors and ranged from loose to dense connective tissue (Figs. 2b, 2c and 3a). Some cases exhibited pseudoangiomatoid spaces due to mucoid change of intercellular matrix with an endothelial-like inner cell layer around the mucoid material (Figs. 2a and 3b). In five cases, besides the spindle cell tumor, an adenomyoepithelioma was found, which probably represented the lesion of origin. In other four tumors, foci of squamous differentiation not exceeding 10 % of tumor cells were seen (Fig. 2c). Both features appeared not to be associated with higher risk of recurrence. In metastasis, the spindle cell morphology and immunophenotype were retained but the cellularity appeared to be denser than in the original tumor (Figs. 2d and 3d). Tumor cells were classified G1 in 10 cases (23.8 %), G2 in 24 cases (57.1 %), and G3 in eight cases (19 %). The rate of events was similar in the G1 and G3 groups. Mean disease-free and mean overall survival were longer in the G1 group compared to the G3 group (29 vs.10 months and 69 vs. 57 months), but the difference was not statistically significant (Table 2).

The Ki-67 labeling index ranged from 5 to 70 % (median 25 %), 19 tumors exhibited a Ki-67 labeling index from 5 to 20 %, 18 tumors from 25 to 40 % (Fig. 3c), and five tumors from 50 to 70 %. A higher Ki-67 labeling index, exceeding 20 %, was associated with a shortened disease-free survival but not overall survival (HR 0.51, log-rank test 0.06; Table 2). All tumors were negative for ER, PR, and HER2. The most frequently expressed myoepithelial markers were CD 10 (Fig. 3b), SM-actin, CK 5–14 (Fig. 3d), and P63/P40 (Fig. 3a), whereas CK 8–18 was negative in the majority of tumors and CD34 was negative in all cases tested (*n* = 15) (Table 3).

**Table 3:** Immunohistochemical features of cases

Antigen	0 % positive cells	−10 % positive cells	≥10 and <50 % positive cells	≥50 % positive cells
CD10 (*n* = 41)	1	0	1	39
CD34 (*n* = 15)	15	0	0	0
CK5/14 (*n* = 42)	1	3	3	35
CK8/18 (*n* = 41)	25	4	6	6
P63 (*n* = 42)	1	0	4	37
P40 (*n* = 40)	1	3	6	30
SM-actin (*n* = 41)	1	1	1	38
EGFR (*n* = 39)	1	4	4	30
ER (*n* = 42)	42	0	0	0
PR (*n* = 42)	42	0	0	0
HER2 (*n* = 42)	42	0	0	0
Ki-67 (*n* = 42)	0	6	31	5

### Molecular findings

Copy numbers for EGFR, c-myc, FGFR, PLAG, and c-met were determined by fluorescence in situ hybridization using tissue microarrays. No significant copy number aberrations were found. Targeted DNA sequencing revealed a high frequency of PIK3CA mutation (18 out of 36 samples; 50 %; mean sequencing depth 4575 reads, range 1135–8128 reads). All mutations were confirmed by Sanger sequencing. The H1047R mutation was the most frequent type (Table 4). PIK3CA mutation altogether and especially the H1047 type tended to be associated with a shorter DFS and OS. In the PIK3CA-mutated group DFS was 14 months and in the PIK3CA-wild type group 34 months (log-rank test *p* = 0.94). With regard to OS, the difference between the two groups was 41 vs. 60 months (log-rank test *p* = 0.241, HR 2.16). In the 12 patients with H1047R mutation, mean DFS was shortened from 35 to 13 months (log-rank test *p* = 0.56, HR 1.38) and OS from 60 to 38 months (log-rank test *p* = 0.15, HR 2.59). All other molecular aberrations assessed in this retrospective study, including TP53, occurred only in a minority of cases or were not associated with OS or DFS. Mutations of TP53 were observed in three out of 30 cases (10 %; K132T; R196Q; P72fs). Mutation of EGFR (G735S and A871T) was found in two cases and of c-met in other two out of 30 cases (E168D and T1010I) (Table 4). No mutations were discovered by targeted sequencing of AKT1, ALK, BRAF, CTNNB1, ERBB2, FBXW7, FGFR2, KRAS, MAP2K1, NRAS, PTEN, and STK11 (represented in both panels). CDH1, FOXL2, GNAQ, GNAS, KIT, MSH6, PDGFRA, SMAD4, and SRC, covered only by the Illumina platform (*n* = 13), and DDR2, ERBB4, FGFR1, FGFR3, and NOTCH1, covered only by the Life Technology platform (*n* = 29), respectively, were also not mutated. Conventional pyro- and Sanger sequencing revealed SF3B1 mutation in two cases (E622K, L707*), whereas other RNA splice factor genes SRSF2 and U2AF1 were not mutated (*n* = 37).

**Table 4:** Gene mutations in SCMC

Gene	Mutation	Number	% of cases studied
*PIK3* CA	None	18/36	50.0
	E545K	2	5.5
	H1047R	12	33.3
	E542K	1	2.7
	V105-108del	2	5.5
	N1068fs	1	2.7
*TP53*	None	27/30	90.0
	K132T	1	3.3
	R196Q	1	3.3
	P72fs	1	3.3
*MET*	None	28/30	93.3
	E168D	1	3.3
	T1010I	1	3.3
*EGFR*	None	28/30	93.3
	G735S	1	3.3
	A871T	1	3.3
*SF3B1*	None	28/30	93.3
	E622K	1	3.3
	L707*	1	3.3

### Types of treatment

Mastectomy was performed in 18 patients, and 23 patients had breast-conserving therapy. In the mastectomy group, both DFS and OS did not differ significantly compared to the less radical procedure (Table 2). Tumor-free margins were indicated by the referring pathologist in 29 cases and unknown in 13 of the surgical specimens (mastectomies in eight cases). Tumor-free margins ranged from 0 to 25 mm (median 3 mm). Tumor tissue reached the sample margin in two cases (“0 mm”), but the R0 situation was affirmed by the surgeon. Patients who were resected with a surgical margin < 5mm were compared with those who had a margin > 5mm (Table 2). Event rates were similar and no significant difference was found regarding DFS and OS. Axillary dissection was conducted in 11 patients and had no significant influence on DFS or OS. Twenty-three patients had no adjuvant therapy, 10 received radiotherapy alone, and seven patients had combined radio-/chemotherapy. In two patients, no information on adjuvant therapies was available.

In those patients who had received combined radio- and chemotherapy, no local recurrence, no metastasis, and no death occurred. Radiotherapy and the combined radio- and chemotherapy were compared to no treatment with respect to DFS or OS (log-rank test; DFS *p* = 0.0507, OS *p* = 0.0715) (Table 2). In a pairwise comparison, only the combination of radio- and chemotherapy had a statistically significant influence (log-rank test; DFS *p* = 0.015, OS *p* = 0.079) (Table 2).

For multivariate analysis, a multivariable regression model was set up with types of treatment (BCT vs. mastectomy), adjuvant therapies and PIK3CA as independent covariables. In a stepwise (entry criterion *p* < 0.25, retaining criterion *p* < 0.2) and a backward (retaining criterion *p* < 0.2) model selection process, none of the covariables was retained in either model.

## Discussion

Rare types of breast cancer usually provide a challenge because no prospective therapy trials, as in breast cancer of no special type, are available. Data from studies on breast cancer of no special type cannot simply be extrapolated and transferred to the rare subtypes. MBC represents a rare subtype and is in itself heterogeneous. Among MBC, there is a subgroup of pure spindle cell tumors with a myoepithelial-like phenotype (SCMC) [3–6]. It has been reported previously that, despite their benign appearance, spindle cell carcinoma of the breast shows aggressive behavior and progresses in a fashion similar to that of invasive duct carcinomas of no special type [8, 9, 11, 12]. The retrospective series presented here confirms the potentially aggressive course. Cumulative event rate regarding local recurrence or metastasis amounted to 39.0 %, and the cumulative event rate regarding death of any cause reached 29.2 %. In addition, a finding made previously by Oberman et al. [10] was confirmed by this study. Conventional histopathological parameters, such as grade, mitotic activity, and presence of squamous epithelium, were not related to outcome (Table 2), although it cannot be excluded that the number of cases was too small to demonstrate a significant association. Missing or low rate of nodal metastases, as found in this series, was also reported by others [8, 11, 13]. Although we present one of the largest series of this special type of metaplastic breast cancer, published so far, the case number was too small to identify significant prognostic markers in the chosen multivariable regression model, and potential prognostic markers retained significance only in the univariate analysis.

Up to now, only little is known about the somatic mutations which give rise to SCMC. Molecular studies conducted on MBC are hampered by the heterogeneity and diversity of MBC. Only rarely have correlations of molecular findings with the histologic subtypes within this rubric of mammary neoplasms been undertaken. Biphasic and monophasic types of MBC have been lumped together [15]. Accordingly, it was found that MBC constitutes a heterogeneous group of tumors also in terms of their gene copy number aberrations and transcriptomic profiles [16]. Genetic changes of Wnt pathway genes have been found as common events in metaplastic carcinomas of the breast [17]. This could not be confirmed by Lacroix-Triki et al. [18] who identified no *CTNNB1* mutation in any of the 21 metaplastic carcinomas. A high copy number of EGFR via aneusomy has been described [19]. Overexpression of EGFR by MBC is partly caused by gene amplification without evidence of activating mutations within the *EGFR* gene [20]. In contrast to other types of MBC, the subtype under study here did not show *EGFR* gene amplification, and *EGFR* gene mutation rarely occurred (*n* = 2). Frequent aberrations of the *p53* tumor suppressor gene characterize metaplastic breast cancer [15, 21]. In the case of bimodal differentiation, both components shared p53 mutation indicating that the gene is altered at an early stage of clonal expansion [15, 21]. In SCMC, *p53* mutation appears to be less frequent and we observed *p53* mutation in only 10 % of cases.

The PI3K catalytic isoform p110α is the second most frequently mutated oncogene. It belongs to the PI3K/AKT/mTOR (phosphatidylinositol 3 kinase/Akt/mammalian target of rapamycin) signaling cascade, which is downstream of EGFR and plays a crucial role in diverse cellular processes such as cell growth, survival, proliferation, and migration. PI3K mutation proved to be the most frequent somatic mutation detectable in breast cancer, particularly in luminal breast cancer [23, 26]. By contrast, it seems to be considerably rarer in the basal type or triple negative type [23]. In this respect, the myoepithelial-like spindle cell metaplastic carcinoma seems to be more similar to the luminal type, with a high proportion of *PI3KCA*-mutated cases ranging to 50 %. As novel agents antagonizing mutated *PI3KCA* enter clinical trials and show encouraging clinical activity, in particular with regard to the H1047 mutation [28], the blockade of the *PI3K*/*AKT*/*mTOR* pathway could be a potential treatment option for this subtype of MBC. Luminal expression of *PIK3CA* mutant H1047R in mice induced adenomyoepithelial-like tumors [29] which could partly reflect the pathogenesis of myoepithelial-like spindle cell MBC, which can evolve from intraductal adenomyoepithelial tumors. In concordance with this speculation, intraductal papilloma to which adenomyoepithelial tumors are related revealed a high frequency of *PI3K*/*AKT* mutation [30].

Whereas all patients in this series received surgical therapy, only a minority (*n* = 7) was treated by chemotherapy. A potential reason for the restraint of clinicians to perform adjuvant chemotherapy may be given by the histologically low-grade aspect of SCMC and the lack of study data on this rare type of MBC. Although the number of adjuvantly treated patients in our series of SCMC is small, there is significant evidence that SCMC patients might benefit from adjuvant chemotherapy. A potential benefit of adjuvant chemotherapy was also noted in a large retrospective study including all types of MBC [31]. Currently, there are no reliable criteria available to identify the high-risk subgroup among SCMC which will progress and metastasize. Further molecular investigations beyond the genes analyzed in this study may uncover molecular markers of progression. The frequent activation of the PI3K/AKT/mTOR pathway by PI3K mutation in SCMC might represent a druggable genetic aberration.
